# Modulation of Stiffness-Dependent Macrophage Inflammatory Responses by Collagen Deposition

**DOI:** 10.1021/acsbiomaterials.3c01892

**Published:** 2024-03-11

**Authors:** Vijaykumar S. Meli, Andrew T. Rowley, Praveen K. Veerasubramanian, Sara E. Heedy, Wendy F. Liu, Szu-Wen Wang

**Affiliations:** † Department of Biomedical Engineering, University of California Irvine, Irvine, California 92697, United States; ‡ UCI Edwards Lifesciences Foundation Cardiovascular Innovation and Research Center, University of California Irvine, Irvine, California 92697, United States; § Department of Chemical and Biomolecular Engineering, University of California Irvine, Irvine, California 92697, United States; ∥ Department of Molecular Biology and Biochemistry, University of California Irvine, Irvine, California 92697, United States; ⊥ Institute for Immunology, University of California Irvine, Irvine, California 92697, United States; # Chao Family Comprehensive Cancer Center, University of California Irvine, Irvine, California 92697, United States

**Keywords:** collagen, immune cell, inflammation, LAIR-1, YAP, substrate stiffness

## Abstract

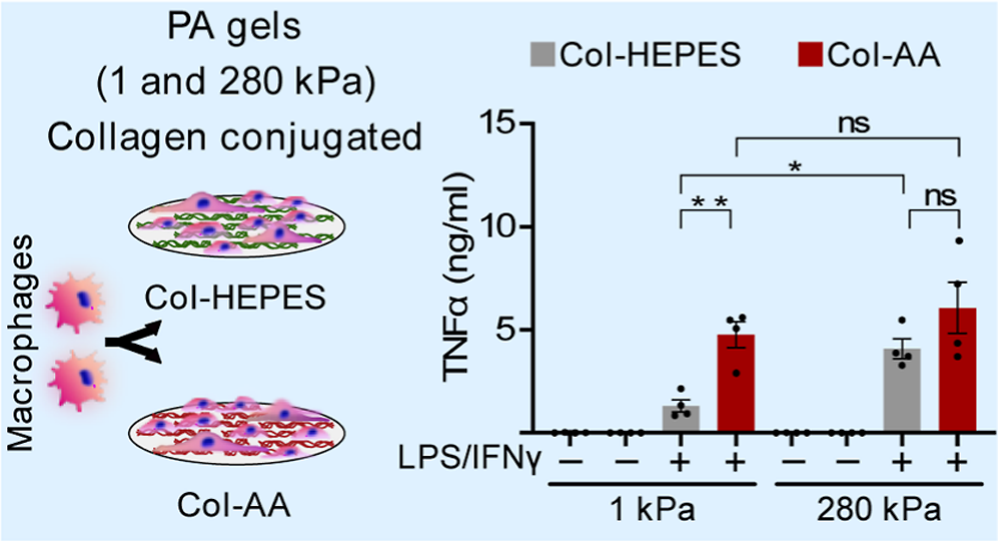

Macrophages are innate immune cells that interact with complex extracellular matrix environments, which have varied stiffness, composition, and structure, and such interactions can lead to the modulation of cellular activity. Collagen is often used in the culture of immune cells, but the effects of substrate functionalization conditions are not typically considered. Here, we show that the solvent system used to attach collagen onto a hydrogel surface affects its surface distribution and organization, and this can modulate the responses of macrophages subsequently cultured on these surfaces in terms of their inflammatory activation and expression of adhesion and mechanosensitive molecules. Collagen was solubilized in either acetic acid (Col-AA) or *N*-(2-hydroxyethyl)piperazine-*N′*-ethanesulfonic acid (HEPES) (Col-HEP) solutions and conjugated onto soft and stiff polyacrylamide (PA) hydrogel surfaces. Bone marrow-derived macrophages cultured under standard conditions (pH 7.4) on the Col-HEP-derived surfaces exhibited stiffness-dependent inflammatory activation; in contrast, the macrophages cultured on Col-AA-derived surfaces expressed high levels of inflammatory cytokines and genes, irrespective of the hydrogel stiffness. Among the collagen receptors that were examined, leukocyte-associated immunoglobulin-like receptor-1 (LAIR-1) was the most highly expressed, and knockdown of the *Lair-1* gene enhanced the secretion of inflammatory cytokines. We found that the collagen distribution was more homogeneous on Col-AA surfaces but formed aggregates on Col-HEP surfaces. The macrophages cultured on Col-AA PA hydrogels were more evenly spread, expressed higher levels of vinculin, and exerted higher traction forces compared to those of cells on Col-HEP. These macrophages on Col-AA also had higher nuclear-to-cytoplasmic ratios of yes-associated protein (YAP) and transcriptional co-activator with PDZ-binding motif (TAZ), key molecules that control inflammation and sense substrate stiffness. Our results highlight that seemingly slight variations in substrate deposition for immunobiology studies can alter critical immune responses, and this is important to elucidate in the broader context of immunomodulatory biomaterial design.

## 1 Introduction

Macrophages are innate immune cells that encounter a variety of complex extracellular matrix (ECM) environments that vary in their biophysical and biochemical properties.^1^ Among the many ECM proteins, collagen is the most abundant in the body^2−4^ and plays a critical role in regulating immune cell function.^4−6^ Collagen maintains structural integrity of the ECM architecture in most connective tissues and contributes to different morphologies and functions of interacting cells.^7^ Scaffolds derived from collagen have been extensively used as biomaterials for tissue engineering due to their biocompatibility, low immunogenicity, permeability, and ability to form highly organized three-dimensional structures.^7−9^

However, tissue damage either due to infection or injury can instigate degradation of collagen that enhances inflammation and initiates an immune response or tissue repair.^10^ Furthermore, alterations in collagen composition and structure have been associated with many diseases and disorders^11^ including cancer, where collagen forms a major component of the tumor microenvironment and promotes disease progression.^12^ Interestingly, macrophage and other myeloid cell interactions with collagen are thought to be involved in immune evasion and cancer progression.^12,13^ Overall, a deeper understanding of macrophage interactions with collagen will be valuable for developing improved tissue engineering strategies and an understanding of health and disease.

Immune cells express receptors that recognize and bind to specific regions in the collagen protein and lead to modulation of their function. These receptors include integrins, discoidin domain receptors (DDRs), mannose receptors, and leukocyte-associated immunoglobulin-like receptor-1 (LAIR-1 or CD305).^14^ In our earlier work, we showed that culturing macrophages on reconstituted collagen hydrogels or surfaces conjugated with a LAIR-1 ligand peptide (sequence derived from collagen III) resulted in suppression of inflammatory activation after lipopolysaccharide (LPS) treatment.^15−17^ In addition, we have demonstrated that biomaterial stiffness modulates macrophage function, with increased stiffness leading to enhanced inflammatory responses.^17,18^ However, these latter studies were performed with hydrogels coated with fibronectin and fibrinogen, specialized matrix proteins that are more abundantly expressed during wound repair, and the effects of stiffness on macrophage–collagen interactions were not examined.

Collagen type I is commonly used as a ligand on hydrogel substrates to encourage cell adhesion and is often coated using different solvents. Although collagen is more soluble in acidic solvents, most of the established protocols to coat the hydrogel surfaces with collagen type I use neutral or basic solvents.^19^ Fibrillar collagen (e.g., type I) is formed by the self-assembly of tropocollagen units into small fibrils and then to larger fibers.^2^ Earlier studies have demonstrated that collagen I coating on the substrate depends on the solvent type and pH;^20^ collagen at low pH forms soluble trimers, while higher-order macromolecular assemblies and fibrils are observed at high-pH conditions.^21,22^ Furthermore, using acetic acid in the solvent (low pH) leads to a higher efficiency of surface attachment and homogeneous distribution.^20,23^ Despite these variations resulting from different coating protocols and solvents, the cell response to the varying collagen distribution of the hydrogel surface is not typically considered. There is evidence, however, that such details are important to evaluate. For example, when mesenchymal stem cells (MSCs) were cultured on substrates that were coated with collagen solubilized in acetic acid, the cells exhibited more cell spreading and enhanced YAP nuclear translocation (relative to cells grown on collagen deposited at other pH).^2,20^ Immune cells, particularly macrophages, interact with collagen under physiological and pathological conditions, but the effects of different collagen structures and substrate stiffnesses on macrophages have not been explored.

Here, we show that conjugating collagen to surfaces using different solution conditions, which are typically used for depositing collagen on substrates (acetic acid pH 3.4 and *N*-(2-hydroxyethyl)piperazine-*N′*-ethanesulfonic acid (HEPES) pH 8.5), can ultimately influence the inflammatory activation of macrophages that are cultured on these surfaces and override the effect of biomaterial stiffness. Collagen was solubilized in either acetic acid or HEPES solutions and conjugated onto soft or stiff polyacrylamide (PA) hydrogel surfaces, and the resulting surfaces were washed well with cell culture media to remove residual conjugation reagents. PA hydrogels were examined that span the physiological stiffness range of typical tissues (1–280 kPa). Murine bone marrow-derived macrophages (BMDMs) that were cultured on PA hydrogels exhibited different levels of inflammatory activation, depending on whether the surface was conjugated with collagen that was solubilized in solutions with acetic acid (Col-AA) or HEPES (Col-HEPES). Further, we investigated the expression of collagen receptors in macrophages cultured on functionalized surfaces and examined the inflammatory activation of macrophages after knocking down the expression of one of the major collagen receptors. Next, we determined how solvents at different pH affect collagen distribution and subsequent cell spreading, focal adhesions, and traction force experienced by the macrophages. Finally, we probed the key mechanosensitive molecules that can control inflammation in macrophages cultured on Col-HEP and Col-AA surfaces.

## 2 Materials and Methods

### 2.1 PA Hydrogel Synthesis and Collagen Surface Attachment

PA hydrogels with tunable mechanical properties were synthesized on glass coverslips according to a previously described protocol.^19^ The stiffnesses of the PA gels were determined and characterized previously.^19,24^ To prepare surfaces for PA hydrogels, 18 mm glass coverslips were soaked in 100% ethanol and sonicated for 10 min to clean, air-dried, and ultraviolet–ozone (UVO) treated for 10 min. Bind-silane solution (95% ethanol + 0.3% 3-(trimethoxysilyl) propyl methacrylate and 5% of 10% acetic acid) was added on the surfaces for 5 min, then blot-dried, and placed at ∼70 °C for 1 h. To make the PA gels, 40% acrylamide, 2% bis-acrylamide, and phosphate-buffered saline (PBS) were mixed, and then, ammonium persulfate and *N*,*N*,*N*,*N*′-tetramethylethylenediamine (TEMED) were added. In addition to the coverslips, hydrophobic glass slides were made by treating the surface with silanization solution I (Sigma-Aldrich) for 5 min. The gel solution was added on the surface of the silanated glass slide, and the bind-silane-treated coverslips were placed on top of the gels contacting the gel so that the PA gel was sandwiched between the hydrophobic surface of the glass slide and the bind-silane-treated surface of the coverslip. After 30 min, the coverslip was separated from the glass slide and the surface rinsed with PBS. To conjugate collagen, the PA-coated glass coverslips were treated with cross-linking agent sulfo-SANPAH (Thermo Scientific) under UV for 10 min. Then, the rat tail 100 μg/mL collagen I (Corning) that was solubilized in aqueous solutions of either 50 mM HEPES buffer (HEPES) (pH, 8.5) or 0.05% acetic acid (AA) (pH, 3.4) was incubated with the PA-coated coverslips overnight at 4 °C. The collagen-conjugated gels were then washed three times with the cell culture media before culturing with cells to remove the residual solvent (HEPES or AA) and unbound collagen.

### 2.2 Isolation and Differentiation of BMDMs

All studies requiring animals were carried out according to protocols approved by the Institutional Animal Care and Use Committee (IACUC) at the University of California, Irvine, which is fully accredited by AAALAC. Bone marrow cells were flushed from femurs of 6–12 week old female C57BL/6J mice (Jackson Laboratories). The isolated cells were treated with ACK lysing buffer to remove any red blood cells (Thermo Fisher Scientific), and subsequently cultured in media consisting of Dulbecco’s modified Eagle’s medium (DMEM) (pH 7.4) supplemented with 10% heat-inactivated fetal bovine serum (FBS), 2 mM l-glutamine, 1% penicillin/streptomycin (all components from Thermo Fisher), and 10% conditioned media with macrophage colony-stimulating factor (M-CSF) (macrophage culture media). The cells were fed with the same media on day 3 and dissociated from the culture plate on day 6 using an enzyme-free cell dissociation buffer (Thermo Fisher Scientific). All of the experiments with the BMDMs were performed using freshly differentiated cells.

### 2.3 THP-1 Cell Culture and Differentiation

THP-1 (TIB-202) cells were obtained from the American Type Culture Collection (ATCC) and cultured using ATCC-formulated RPMI-1640 medium (Cat. no. 30-2001) supplemented with 0.05 mM 2-mercaptoethanol and 10% FBS. The cells were maintained in a suspension at a concentration of 2–4 × 10^5^ cells/mL. To differentiate the THP-1 monocytes from macrophages, 20 nM phorbol myristate acetate (PMA) was added to the media for 42 h before any experiments. All of the experiments were performed using THP-1 cells with passages up to 12.

### 2.4 Assessment of Cytokine Secretion by ELISA

After 6 days of culture with media containing M-CSF, BMDMs were dissociated from the plate using cell dissociation buffer and seeded on different stiffness PA hydrogels coated with collagen-I using HEPES and AA buffer. The hydrogels were first placed in a 24-well culture plate, and the cells were seeded on top of these gels at a density of 0.1 million cells/well using 400 μL of the media. After 24 h of culture, the cells were stimulated with 0.5 ng/mL ultrapure LPS (InvivoGen) and 1 ng/mL interferon gamma (IFNγ) (R&D Systems) in 100 μL of the media (M1 stimulation). After 24 h of stimulation, the cell supernatants were collected for assessment of cytokine secretion by enzyme-linked immunosorbent assay (ELISA) following the manufacturer’s protocol (Biolegend).

### 2.5 Immunofluorescence Staining and Quantification

For immunostaining, the cells were immediately fixed in 4% PFA (paraformaldehyde; Electron Microscopy Sciences) for 10 min at room temperature (RT) and were washed 3 times with PBS (Thermo Fisher Scientific) and permeabilized for 10 min using 0.3% Triton X-100 in PBS. Samples were then blocked with 2% bovine serum albumin (BSA) in PBS for 1 h at RT. The samples were incubated in the following primary antibodies overnight at 4 °C: collagen (COLA1 from ABclonal), inducible nitric oxide synthase (iNOS) (Abcam), LAIR-1 (Thermo Fisher Scientific), YAP (Santa Cruz Biotechnology), or transcriptional co-activator with PDZ-binding motif (TAZ) (Santa Cruz Biotech.) antibodies. Cells were then washed with 2% BSA in PBS and incubated with secondary antibody (Alexa Fluor 594 antirabbit IgG antibody (Biolegend)) at RT for 1 h. Nuclei and actin were stained using Hoechst and Alexa Fluor 594-phalloidin (Invitrogen), respectively, diluted in 2% BSA in PBS for 30 min at RT. Finally, the cells were washed three times with PBS and mounted on glass slides using Fluoromount G (Southern Biotech). Images were acquired at 40× using an Olympus FV3000 laser scanning confocal microscope using the software FLUOVIEW FV3000. The images captured were analyzed using ImageJ software to analyze fluorescence intensity and YAP/TAZ nucleo-cytoplasmic localization. Briefly, cell boundaries were outlined manually using actin stain, and the nuclear boundaries were defined using the Hoechst stain. Using ImageJ, the integrated density of the region of interest was measured, and the area around the cell was used as background. The total nuclear intensity of YAP/TAZ was divided by the total intensity of YAP/TAZ in the cytoplasm to obtain the nuclear-to-cytoplasmic ratio.

### 2.6 Immunoblot Analysis

After 24 h of M1 stimulation, the BMDMs were lysed using radioimmunoprecipitation assay (RIPA) lysis buffer (VWR) supplemented with 1× Halt protease and phosphatase inhibitor cocktail (Thermo Fisher Scientific). Twenty micrograms of total protein was resolved on 4–15% Mini-PROTEAN TGX precast gels (Bio-Rad) and blotted onto nitrocellulose membrane using iBlot2 transfer systems (Invitrogen). The blots were incubated with 1:1000 primary antibodies of anti-iNOS (a marker protein for inflammation) (Abcam) and anti- glyceraldehyde 3-phosphate dehydrogenase (GAPDH), a housekeeping protein, (Santa Cruz Biotechnology) for 1 h at RT in Tris Buffer Saline-Tween-20 (TBST-made with 20 mM Tris, 150 mM NaCl and 0.1% Tween 20), and after three washes with TBST, it was further incubated with horseradish peroxidase–conjugated secondary antibodies (Biolegend) for 1 h. Finally, the blot was incubated in a SuperSignal West Femto Maximum Sensitivity Substrate (Thermo Fisher Scientific) for 5 min before imaging the blot using Bio-Rad ChemiDoc XRS+ with Image Lab software.

### 2.7 RNA Isolation, cDNA Preparation, and qRT-PCR Analysis

For real-time polymerase chain reaction (PCR) measurements, BMDMs were stimulated with M1 cytokines (LPS and IFNγ) and lysed after 4 or 24 h using TRI reagent (mixture of guanidine thiocyanate and phenol in a monophasic solution from Sigma), and RNA was isolated following the manufacturer’s protocol. Approximately 1 μg of RNA was used to synthesize cDNA using the High Capacity cDNA Reverse Transcription Kit from Applied Biosystems. Green SuperMix Reaction Mix (Azura Genomics) was used for quantitative real-time PCR, and a total of 40 cycles were performed on Bio-Rad’s CFX-96 real-time PCR system. Relative gene expression was analyzed by 2^–ΔΔ*CT*^ method^25^ and expressed relative to the housekeeping gene *GAPDH*, and data was normalized to the unstimulated condition for the 1 kPa, Col-HEP surface as this condition results in minimal inflammation of macrophages. The primers used for qPCR in this study were designed using Primer3 Plus program and purchased from Integrated DNA Technologies (IDT), and these are described in Table S1.

### 2.8 Gene Knockdown Using siRNA

Knockdown of LAIR-1 was performed by nucleofection (4D-Nucleofector system, Lonza) using SMARTPool siRNAs (M-057755-01-0005a: mixture of 4 siRNAs) (Horizon Discovery). Briefly, half a million freshly isolated BMDMs were transfected with 100 nM corresponding siRNAs in 20 μL of nucleofection solution. After nucleofection, cells were recovered in D10 complete media (DMEM media containing 10% heat-inactivated FBS + 1% penicillin and streptomycin + 10% M-CSF) for 42 h and stimulated with M1 cytokines (LPS, IFNγ). The cells were immunostained, and the supernatant was analyzed as described above.

### 2.9 Fluorescent Collagen-I Labeling

To quantify the collagen cross-linking on the hydrogel surface, we used Cy5 NHS Ester (Fisher Scientific). Briefly, the rat-tail collagen-I was diluted to 1 mg/mL from the stock using HEPES buffer (pH 8.5), and 10 μL (100 μg/mL) of Cy5 −NHS Ester was added. The reaction was incubated at RT for 20 min with shaking. Then, the collagen was diluted to 100 μg/mL using HEPES and acetic acid buffer and cross-linked to the PA hydrogel overnight at 4 °C. The unbound collagen-Cy5-NHS-Ester was washed with respective buffer, and the hydrogel surface was imaged using Keyence BZ-X810 widefield microscope at the UCI Stem Cell Research Center’s imaging facility. Quantification was performed using ImageJ software.

### 2.10 Scanning Electron Microscopy

For characterization of the nanoscale and microscale structures in the hydrogels, the films were affixed to slotted head pin scanning electron microscopy (SEM) stubs (VWR) using conductive carbon tape and then sputter-coated with 4 nm of iridium using an EMS 150 TS sputter coater (Quorum Tech, UK). Samples were imaged using a FEI Magellan 400 XHR Scanning Electron Microscope (FEI Company, Hillsboro, OR). Secondary electron images were acquired using a voltage of 3 kV, current of 25 pA, and a dwell time of 10.0 μs. Finally, the data collection and analysis were carried out using xT microscope control version 5.0.2.2666 build 2666.

### 2.11 Traction Force Microscopy

PA gel substrates were prepared as described previously, with modifications.^19,26^ Briefly, glass bottom dishes (35 mm) were UVO-treated and functionalized using 0.3% (v/v) 3-(trimethoxysilyl) propyl methacrylate (Sigma-Aldrich). Glass coverslips functionalized with poly d-lysine (0.1 mg/mL; Gibco) were coated with 1:800 (v/v) aqueous dilution of red fluorescent microspheres (0.5 μm, carboxylate modified; Thermo Fisher Scientific). The gels (20 kPa) were prepared by sandwiching polymerizing acrylamide-bis(acrylamide) solution on the functionalized glass bottom dishes with microsphere-coated coverslips. After polymerization, the coverslips were peeled off, and collagen-I (100 μg/mL) was conjugated to the surface of gels with sulfo-SANPAH reagent (Thermo Fisher Scientific). Then 25,000 cells per dish were seeded, and traction force microscopy (TFM) imaging was performed. Briefly, images of the microbeads and the cell location were captured after 24 h after seeding and 24 h of stimulation. The cells were released from the gel surface by adding 0.1% sodium dodecyl sulfate (SDS) solution, and the microbeads and cell location were captured. Fiji software was used to register unaligned images. Subsequently, particle image velocimetry and Fourier transform traction cytometry were performed as previously described.^27^ A custom code was written in Python and IJ1 macro language to batch process the single-cell traction forces.^17,28^ The root-mean-square forces are calculated as the square root of the mean-squared forces associated with the bead displacement by single cells, measured using 24-pixel interrogation windows. At least 60 individual cells per condition were analyzed.

### 2.12 Statistical Analysis

All of the experiments were repeated at least three times with cells from three different individual donors (for BMDM) or passages (for THP-1). Values presented here are the mean ± standard error of the mean. For multiple comparisons, one-way analysis of variance (ANOVA) with Tukey’s post hoc test was used. Two-tailed Student’s *t*-tests were performed for pairwise comparisons. For all the statistical tests, *p*-values less than or equal to 0.05 are designated by single star (*), and *p*-values less than 0.005 are designated by double star (**), and these were considered significant.

## 3 Results

### 3.1 Solvent Systems Used in Collagen Conjugation onto Soft and Stiff Hydrogels Affect Inflammatory Activation of Macrophages

**Figure 1:**
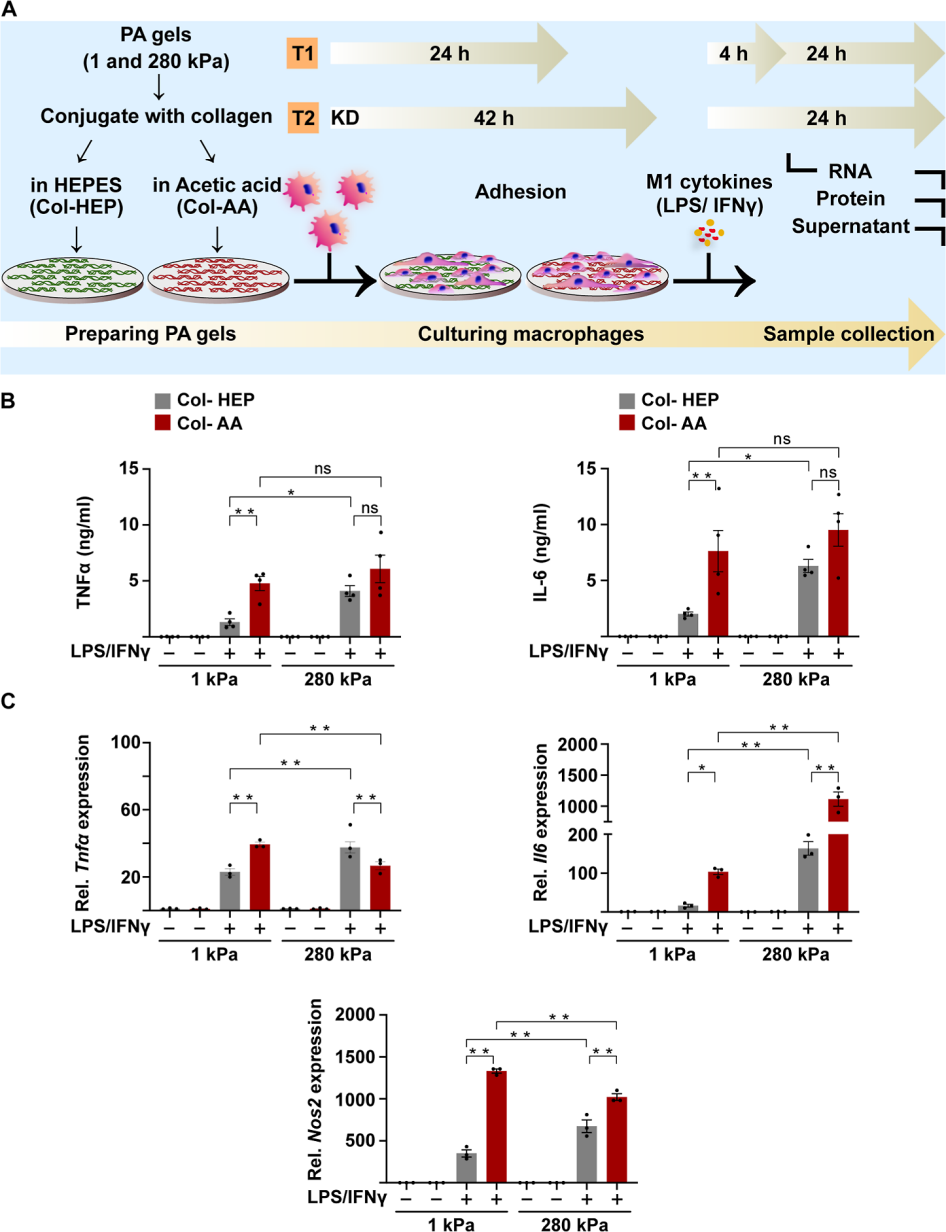
Solution system used for collagen conjugation on PA hydrogels affects inflammatory activation of macrophages. (A) Schematic showing the timeline and the details of experiments. PA hydrogels were conjugated with collagen overnight in HEPES (Col-HEP) or acetic acid (Col-AA) solutions and rinsed well with DMEM. Macrophages were cultured for 24 h and stimulated with M1 cytokines for an additional 4 or 24 h [timeline T1]. For knockdown (KD) experiments, after the siRNA nucleofection, cells were cultured for 42 h and stimulated with M1 cytokines for 24 h [timeline T2]. At the end of the experiments, the supernatant, RNA, and proteins were collected for analysis. (B) Secretion of TNFα and IL-6 by BMDMs cultured on PA hydrogels of 1 and 280 kPa, coated with either Col-HEP or Col-AA, after 24 h of adhesion and 24 h of stimulation. (C) Relative expression of *Tnf*α, *Il-6*, and *Nos2* assessed by quantitative PCR in BMDMs cultured for 24 h and stimulated for 4 h, and normalized to the unstimulated, 1 kPa Col-HEP condition. The values are the mean ± standard error of the mean (SEM) from at least three individuals, assessed by one-way ANOVA with Tukey’s multiple comparisons. *p < 0.05, **p < 0.005.

To understand the effect of the collagen-conjugation solvent on stiffness-dependent inflammatory activation of macrophages, PA hydrogels of varying stiffness (1 and 280 kPa)^19^ were generated, and the surface was functionalized with collagen I in HEPES buffer (pH 8.5; Col-HEP) or acetic acid (pH 3.4; Col-AA). Murine BMDMs were then cultured in macrophage culture media on these hydrogels, without and with M1-cytokine stimulation (LPS and IFNγ). In all the experiments, equal numbers of cells were plated on each condition (PA gels with Col-HEPES and Col-AA). After 24 h, the PA gels were inspected for cell adhesion, and PA gels that were defective and leading to unequal cell numbers were not considered in the study. The experimental timeline is depicted in the schematic (Figure 1A).

We observed that cells cultured on 280 kPa Col-HEP PA gels secreted 2-fold higher amounts of inflammatory cytokines tumor necrosis factor-α (TNF-α) and interleukin-6 (IL-6) compared to cells on the 1 kPa Col-HEP hydrogels, after stimulation with M1 cytokines (Figure 1B). In contrast, cells cultured on Col-AA PA gels secreted similar levels of TNF-α and IL-6 on both soft (1 kPa) and stiff (280 kPa) hydrogels. Interestingly, we found that macrophages cultured on 1 kPa Col-AA hydrogels secreted significantly higher levels of inflammatory cytokines than that by the cells cultured on 1 kPa Col-HEP (Figure 1B). Similar trends were also observed on the stiff hydrogels (280 kPa) but were not statistically significant. We noted that unstimulated macrophages did not secrete any inflammatory cytokines on all surface treatments and stiffnesses tested. To test whether the inflammatory nature of Col-AA-functionalized surfaces was also true for human macrophages, we performed similar experiments in the THP-1 human monocyte/macrophage cell line. Like BMDMs, THP-1 cells cultured on Col-AA secreted higher levels of TNF-α than that of cells on Col-HEP at both stiffnesses (Figure S1A). Together, these data show stiffness-dependent secretion of inflammatory cytokines in macrophages cultured on Col-HEP surfaces but not on Col-AA surfaces.

For these investigations, we showed that the presence of collagen on the surface is necessary for the observed higher inflammatory activity of cells on the Col-AA substrate. Macrophages were cultured on uncoated PA hydrogels that were incubated in HEPES and acetic acid buffers with no collagen. The uncoated PA hydrogels had fewer adhered cells, and inflammatory activation of macrophages was indifferent between HEP and AA. We did not observe stiffness-dependent secretion of inflammatory cytokines, TNF-α and IL-6 (Figure S1B).

The trends observed for cytokine secretion were also recapitulated in gene expression. Similar to cytokine secretion, gene expression of genes for inflammatory cytokines *Tnfa* ( Tumor necrosis factor), *Il6* (interleukin-6), and *Nos2* (nitric oxide synthase 2) was significantly higher in cells cultured on Col-AA than in cells cultured on Col-HEP after 4 h of M1 stimulation (Figure 1C). After 24 h, overall gene expression of inflammatory genes was lower than that at 4 h, but cells cultured on soft Col-AA PA hydrogels showed higher gene expression than cells cultured on Col-HEP for *Il6* and *Nos2* (Figure S2). In some cases, we observed that the inflammatory gene expression in BMDMs cultured on stiff Col-AA hydrogels was lower than that in BMDMs on Col-HEP. This may be due to different transcription dynamics, leading to different peaks of mRNA expression.

**Figure 2:**
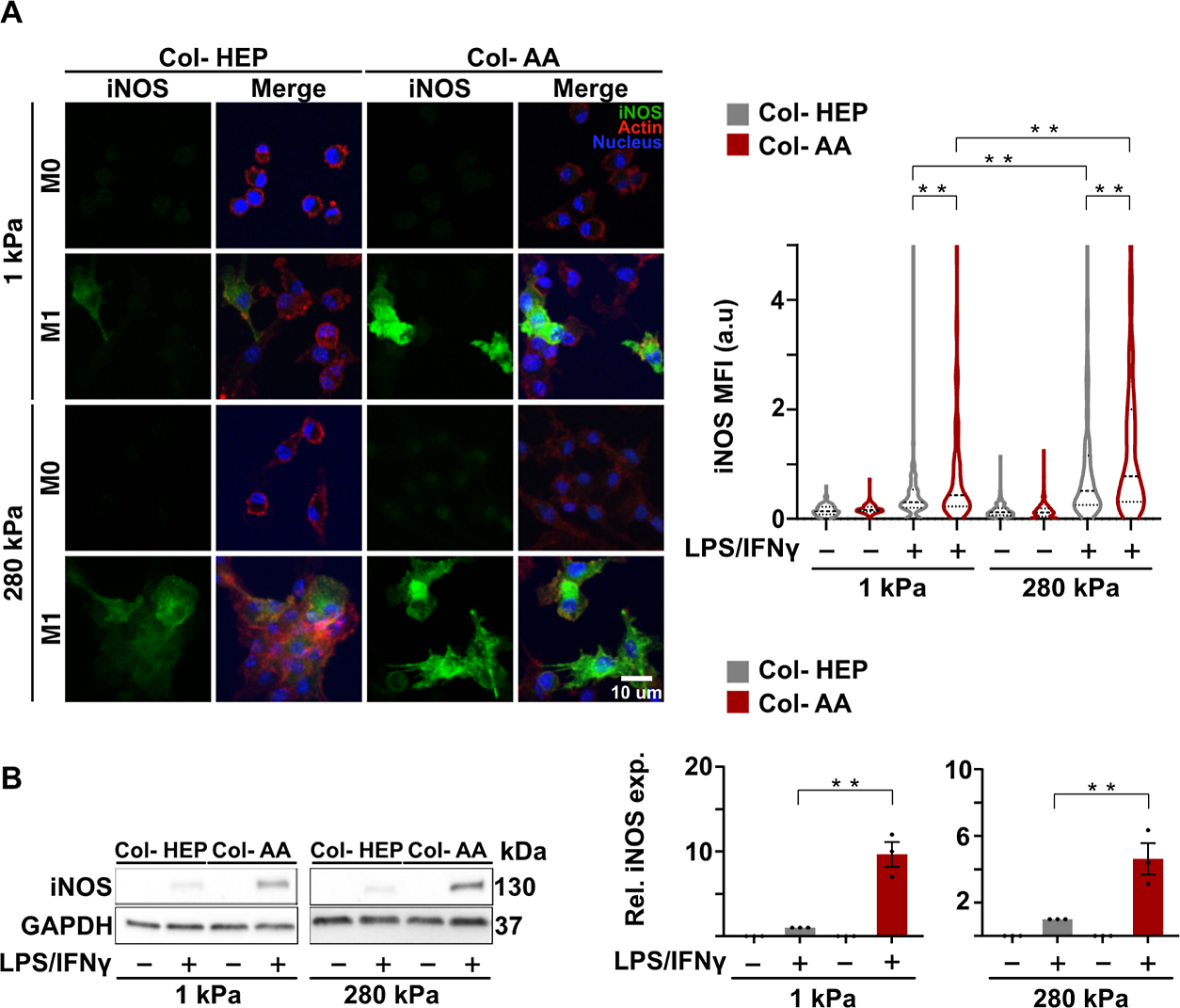
Solvent system used for collagen conjugation on Col-HEP and Col-AA PA hydrogels affects iNOS expression in macrophages. (A) Immunofluorescence confocal images of iNOS (green), F-actin (phalloidin, red), and nuclei (blue) in BMDMs cultured for 24 h on Col-HEP- and Col-AA-conjugated surfaces and stimulated with M1 cytokines for 24 h as shown in Figure 1A. The quantification of iNOS mean fluorescent intensity (MFI) is shown in the plot (right). (B) Immunoblots (left) and quantification (right) of iNOS from cells cultured using conditions described in (A). Values are normalized to those obtained for activated macrophages on Col-HEP surfaces. The values are the mean ± SEM from at least three individuals assessed by one-way ANOVA with Tukey’s multiple comparisons. **p < 0.005. For immunoblots and immunostaining, quantification is an average of three blots or at least 150 cells across three biological replicates, respectively. Violin plots show quartiles and median.

Immunofluorescence staining for intracellular inflammatory marker iNOS showed significantly higher expression in M1-stimulated cells cultured on Col-AA coated hydrogels than in cells cultured on Col-HEP under both soft and stiff conditions (Figure 2A). Consistent with this, immunoblot quantification showed significantly higher expression of iNOS protein in cells cultured on Col-AA coated hydrogels in both soft and stiff conditions compared to that in cells cultured on Col-HEP-coated hydrogels (Figures 2B and S5). Quantitative PCR and immunoblot analyses were normalized to GAPDH, eliminating any differences between groups due to cell numbers. Overall, our results indicate that Col-AA-coated PA hydrogels enhanced the inflammatory activation of macrophages compared with that of Col-HEP-coated hydrogels, irrespective of the substrate stiffness. Furthermore, the inflammatory response is dampened on soft substrates in cells cultured on Col-HEP surfaces.

### 3.2 Knockdown of Collagen Receptor Gene *Lair-1* Enhances Inflammatory Activation of Macrophages

**Figure 3:**
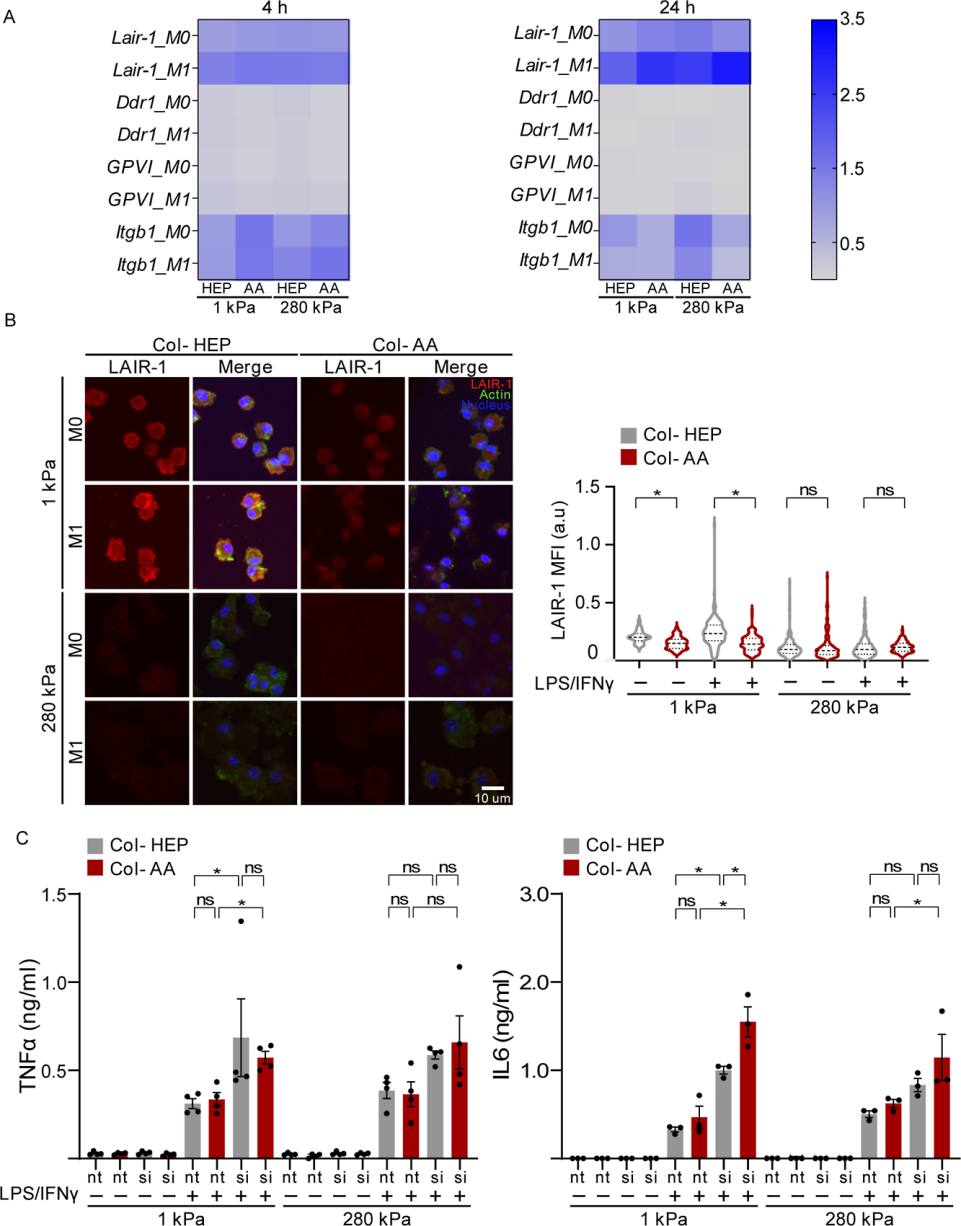
Knockdown of collagen receptor gene *Lair-1* enhances inflammatory activation of macrophages. (A) Relative expression of collagen receptors in BMDMs cultured for 24 h on PA hydrogels of 1 and 280 kPa, with Col-HEP and Col-AA surfaces. Cells were then stimulated with M1 cytokines for 4 or 24 h. (B) Immunofluorescence confocal images of LAIR-1 (CD305) (red), F-actin (phalloidin, green), and nuclei (blue) in BMDMs (left), and quantified LAIR-1 MFI (right) after 24 h of adhesion and 24 h of M1 stimulation. (C) Secretion of TNFα (left) and IL-6 (right) by BMDMs after knockdown using *Lair-1* siRNA (si) or nontarget siRNA (nt; control). Cells were cultured for 42 h and stimulated for 24 h. The values are the mean ± SEM from at least three donors assessed by one-way ANOVA with Tukey’s multiple comparisons. *p < 0.05. For immunostaining, quantification is an average of at least 150 cells across three biological replicates. Violin plots show quartiles and median.

We next investigated the expression of several collagen cell surface receptors and their possible involvement in macrophage inflammatory activation. Known collagen receptors that mediate recognition of triple-helical collagen include integrins, DDRs (e.g., DDR1, encoded by *Ddr1* gene), LAIR-1 (encoded by *Lair1* gene), and glycoprotein VI (*GPVI*).^14^ After culturing the BMDMs on PA hydrogels conjugated with collagen I for 24 h and stimulated with M1 cytokines for 4 and 24 h (Figure 1A), the cells were analyzed for gene expression of collagen receptors. *Lair1* was the most highly expressed gene among the receptors analyzed at 4 and 24 h after stimulation, followed by the integrin beta-1 (*Itgb1*) receptor, and very little expression for *Ddr* and *GPVI* (Figure 3A). Therefore, we focused our subsequent characterization on *Lair1*. The *Lair1* gene expression was similar in cells cultured on both Col-AA- and Col-HEP-coated PA hydrogels, but immunofluorescence staining of the LAIR-1 (CD305) receptor generally showed higher protein expression in cells cultured on softer PA hydrogels relative to the stiff hydrogels (Figure 3B). In cells cultured on soft hydrogels, LAIR-1 expression was higher in cells cultured on Col-HEP than that in cells cultured on Col-AA (Figure 3B), which is consistent with lower inflammatory cytokine secretion and lower inflammatory gene expression for Col-HEP surfaces (Figure 1). As expected, the overall levels of actin staining by phalloidin were relatively low as macrophages do not form prominent stress fibers or actin bundles when cultured on hydrogels.^17^ Furthermore, knockdown of the *Lair-1* gene using small interfering RNA (siRNA) significantly enhanced the secretion of inflammatory cytokines TNF-α and IL-6 by cells cultured on soft PA hydrogels, and this trend was also observed for the stiff surfaces (Figure 3C). However, the effects of the Col-AA-functionalized surface compared to Col-HEP hydrogels in inflammatory activation was not discernible after gene knockdown, even in the nontarget condition, likely due to nonspecific effects of the knockdown procedure (Figures 3C and 1B). Although the reason for this discrepancy is not clear, one possibility is that it may be due to the stress experienced by the cells during siRNA treatment, which involves electroporation and a lengthy timeline for optimal knockdown of the gene (Figure 1A). Taken together, these results in Figure 3 demonstrate that LAIR-1 is a highly expressed collagen receptor, and knocking down the *Lair-1* receptor, an inhibitory receptor for immune cell activation,^15,16^ enhanced the secretion of inflammatory cytokines.

### 3.3 Solvent Conditions Used for Collagen Coating Cause Different Collagen Distribution on Hydrogel Surfaces

**Figure 4:**
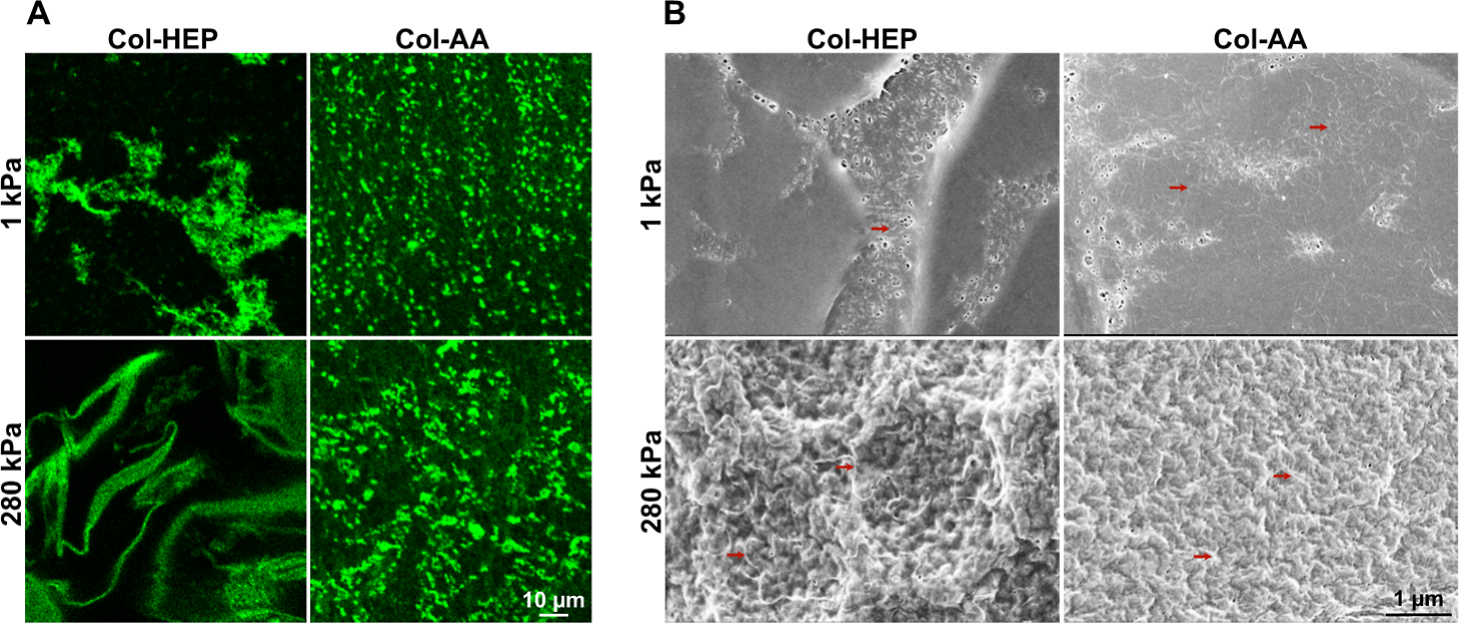
Collagen conjugation solvent affects the collagen distribution on hydrogel surfaces, and cell interaction with the substrate. (A) Immunofluorescence confocal images of collagen (green) on PA hydrogels of 1 and 280 kPa. Surfaces were coated with Col-HEP and Col-AA overnight at 4 °C. (B) SEM images of PA hydrogel surfaces conjugated with Col-HEP and Col-AA as described in (A). Red arrows show collagen fibers in the SEM images.

To determine whether the differences in the inflammatory activation of macrophages cultured on Col-HEP and Col-AA surfaces were correlated with differences in the collagen structure on the surfaces, we examined the collagen distribution on the hydrogel surfaces. Immunofluorescence staining of collagen attached to the PA surfaces showed that in HEPES, collagen self-assembles into heterogeneous fibers and aggregates on both the soft and stiff hydrogels (Figure 4A). However, conjugating in acetic acid solution caused the collagen to be more homogeneously distributed on the surface (Figure 4A), consistent with previously reported results.^20,23^ Because the anticollagen antibody used in Figure 4A was made against a small epitope of collagen, we also examined distribution by labeling the collagen with Cy5 NHS-ester prior to attachment to the hydrogel surfaces (Figure S3A). Data from the Cy5-labeled collagen was consistent with the data acquired with immunofluorescence and confirmed that collagen is heterogeneously distributed on the surface when conjugated in HEPES buffer and more homogeneous on the surface when acetic acid was used as the solvent.

Due to this disparity in the collagen distribution between Col-HEP and Col-AA, we also verified the total amount of collagen on the different hydrogel surfaces using fluorescent imaging to quantify the amount of collagen on these surfaces. We found that depositing with a 100 μg/mL solution of Col-AA yielded a significantly higher amount of conjugated collagen than using a 100 μg/mL solution of Col-HEP surfaces on the soft hydrogels (Figure S3B), consistent with previously published data.^20^ Quantification of images showed that incubating with 50 μg/mL of Col-AA yielded a similar amount of surface-bound collagen as using a 100 μg/mL Col-HEP solution (Figure S3B,C). We found that conjugating 50 μg/mL of collagen on Col-AA surfaces elicited similar inflammatory cytokine secretion (TNF-α and IL-6) by macrophages as that of 100 μg/mL of Col-HEP (Figure S4A); therefore, the differences in total surface collagen concentrations between Col-AA vs Col-HEP are not the reason for the differences in immune activation. Rather than the amount itself, the observed differences are likely due to collagen distribution leading to changes in accessibility of cell binding domains.^20^ We also believe that higher collagen amount on Col-AA surfaces would not lead to stiffness changes that impact the cell behavior as our previous studies on macrophage mechanotransduction^17,29^ suggests that there is stiffness threshold below/above which the cell behavior is unaffected. Therefore, minor changes in stiffness caused by a molecular layer of collagen would not likely have an effect.

To further examine the collagen distribution and the interaction with cells, we performed SEM of the Col-HEP and Col-AA surfaces. The Col-AA surfaces showed a fairly homogeneous distribution of thin fibrils (Figure 4B), consistent with prior data showing surfaces covered with a uniform layer of triple-helical collagen that was deposited at low pH;^23^ however, Col-HEP surfaces showed fewer individual collagen strands and a greater number of large aggregates (Figure 4B). In addition, we made similar observations when macrophages were cultured on Col-HEP and Col-AA surfaces and imaged with SEM (Figure S4B); on Col-AA surfaces, collagen fibrils were generally thinner but more frequent, with increased cell spreading. However, these differences were not very prominent, likely due to deposition of serum proteins during cell culture and harsher treatment of the samples for SEM. Together, these results suggest that collagen was homogeneously distributed on Col-AA surfaces, which likely helped cells to interact with the underlying ECM-based substrate.

### 3.4 Cells on Col-AA Surfaces Exhibit Increased Focal Adhesions, Higher Spread Areas, and Increased Traction Forces Than Cells on Col-HEP

**Figure 5:**
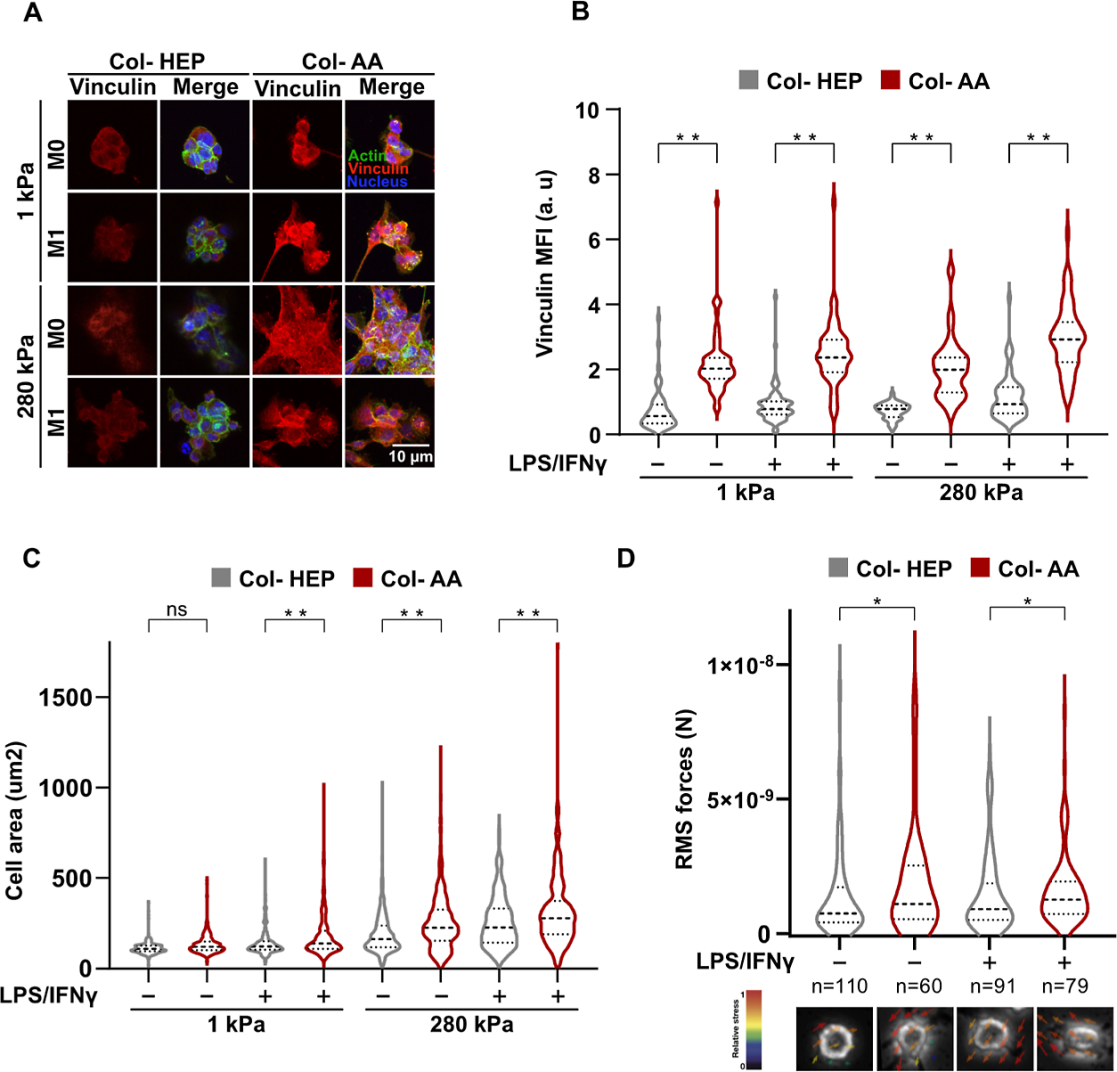
Cells cultured on Col-AA surfaces exhibit larger focal adhesions, higher spread areas, and higher traction forces than those of cells on Col-HEP. (A) Immunofluorescence confocal images of vinculin (red), F-actin (phalloidin, green), and nuclei (blue) in BMDMs cultured onto Col-HEP- and Col-AA coated PA hydrogels of 1 and 280 kPa. Cells were incubated for 24 h, then stimulated with M1 cytokines for 24 h. (B) Vinculin (MFIs) was quantified from confocal images under conditions described in (A). (C) Cell spread areas of BMDMs cultured and stimulated as described in (A). (D) Quantification of root-mean-square (rms) forces and representative bead displacement vectors from particle image velocimetry analysis by TFM (below) by BMDMs on Col-HEP and Col-AA surfaces on PA hydrogels. Cells were adhered for 24 h onto 20 kPa PA hydrogels coated with Col-HEP and Col-AA and stimulated with M1 cytokines for 24 h. *p < 0.05, **p < 0.005. For TFM, at least 60 cells per condition were analyzed. For cell spread, at least 250 cells were analyzed across five biological replicates. For immunostaining, quantification is an average of at least 150 cells across three biological replicates. Violin plots show quartiles and median.

Since the distribution of collagen molecules on Col-AA and Col-HEP surfaces was distinct, we hypothesized that the different surfaces might also alter the cellular adhesion structures that interact with the matrix. Earlier studies have shown that cells spread more on stiffer substrates and have larger focal adhesions and generate increased traction forces.^24,30^ Furthermore, substrate stiffness and contractility are known to influence vinculin recruitment to focal adhesions.^31,32^ In our study, immunofluorescence confocal images of vinculin staining showed higher-intensity staining in macrophages cultured on Col-AA than in those cultured on Col-HEP, for both soft and stiff hydrogels (Figure 5A,B). Further, quantification of the spread cell areas showed that macrophages on Col-AA were more spread than those in the cells on Col-HEP on both soft and stiff substrates (Figure 5C). These data suggest that cells form stronger and more well-established adhesions on Col-AA surfaces and could potentially exert more forces in this environment. To test this, we further analyzed the traction forces exerted by cells by TFM (Figure 5D). TFM showed that cells cultured on Col-AA hydrogels exerted significantly higher traction force than that in cells cultured on Col-HEP. Taken together, these results show that macrophages on Col-AA-conjugated surfaces have higher cell spread area, larger focal adhesions, and exert higher traction forces compared to those of cells cultured on Col-HEP hydrogels. These observations suggest that even on the soft Col-AA surfaces, macrophages might be able to enhance the activation of mechanosensitive as well as inflammatory factors, thus elevating the inflammatory potential.

### 3.5 Macrophages Cultured on Col-AA Shows Higher YAP and TAZ Nuclear Translocation

**Figure 6:**
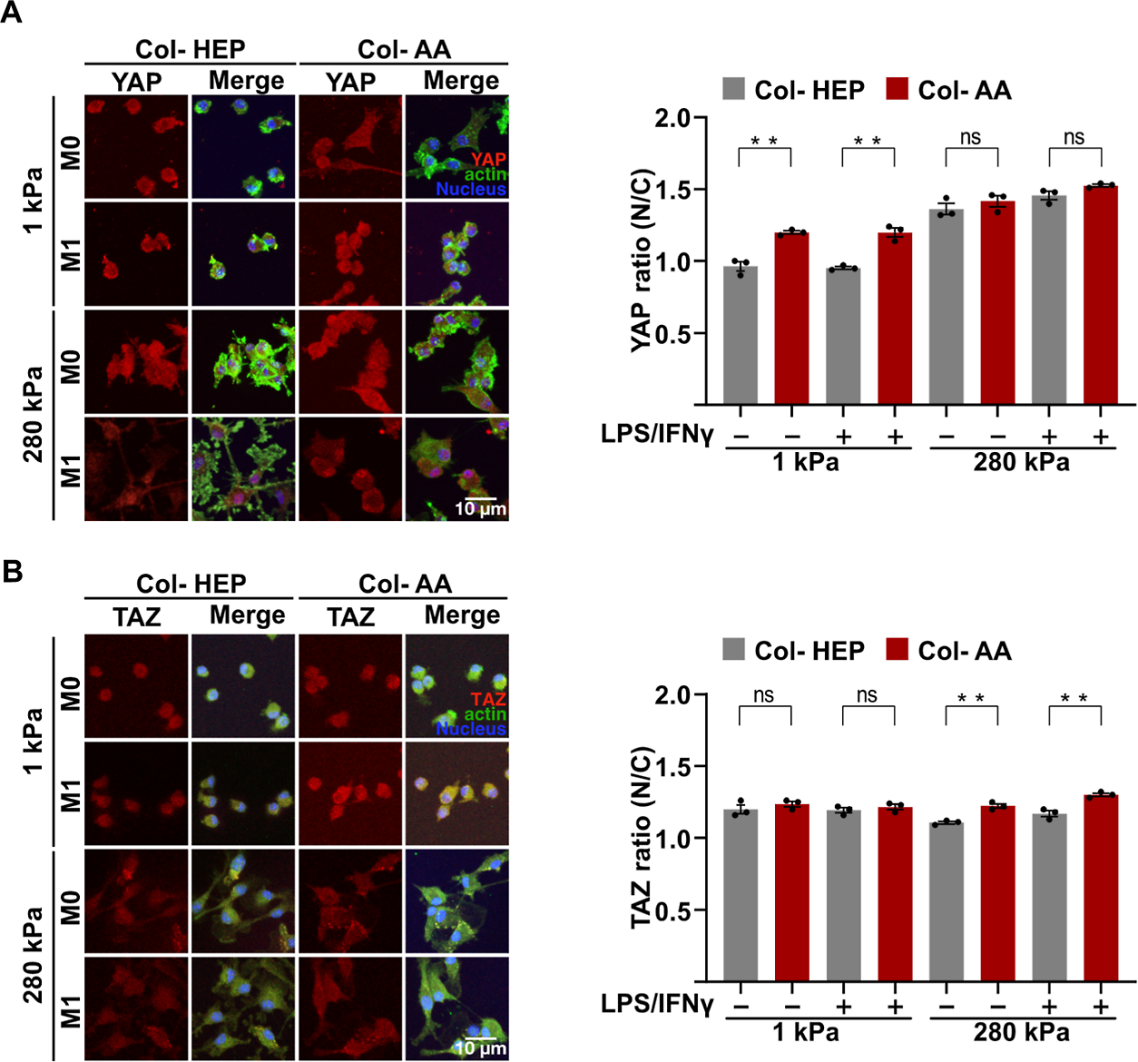
Macrophages cultured on Col-AA show higher YAP and TAZ nuclear translocation. Immunofluorescence confocal images of YAP (A) and TAZ (B) in mouse BMDMs after 24 h of adhesion onto PA hydrogels of 1 and 280 kPa coated with Col-HEP and Col-AA and stimulated with M1 cytokines for 24 h, quantification of nuclear/cytoplasmic (N/C) ratio of YAP (top right) and TAZ (bottom right). The values are the mean ± SEM from at least three donors assessed by two-tailed Student’s *t*-test. **p < 0.005. For immunostaining, quantification is an average of at least 150 cells across three biological replicates.

Greater traction force and higher spread cell have been associated with increased activity of the mechanosensitive cotranscription factor YAP and its paralog TAZ.^33,34^ Moreover, we have demonstrated that the translocation of YAP/TAZ into the nuclei can enhance the inflammatory activation of macrophages.^17^ To examine whether macrophages cultured on Col-AA had a higher nuclear-to-cytoplasmic ratio (N/C) of YAP and TAZ, we stained for YAP and its paralogue TAZ on the cells cultured on Col-HEP and Col-AA. Our results showed that YAP nuclear-to-cytoplasmic ratio was indeed higher in cells cultured on 1 kPa PA Col-AA gels than that of cells cultured on Col-HEP, but the conjugation buffer did not cause differences on the stiff 280 kPa PA gel (Figure 6A). TAZ showed a moderately but significantly higher nuclear-to-cytoplasmic ratio in macrophages cultured on 280 kPa Col-AA gels compared to that of macrophages cultured on Col-HEP, but not in 1 kPa hydrogels (Figure 6B). These results indicate a higher amount of YAP and TAZ translocation to the nucleus in cells cultured on Col-AA compared to that in cells cultured on Col-HEP.

## 4 Discussion

Substrate material properties such as stiffness, composition, and architecture have been shown to influence macrophage inflammatory responses.^1,17,18^ Our earlier work has demonstrated that macrophages cultured on stiffer hydrogels have more spread area and higher secretion of inflammatory cytokines. In addition, we established that different adhesive ECM proteins differentially influence macrophage phenotype and function.^35^ We also demonstrated that transcriptional coactivator YAP in macrophages cultured on stiffer substrates translocates to the nucleus and enhances the inflammatory activation of the macrophages.^17^ In this study, we show that the activation of macrophages cultured on collagen-coated hydrogels depends on the solvent used for conjugation of the matrix protein. Specifically, coating and conjugating PA hydrogel with collagen in an acetic acid solution led to a more homogeneously distributed collagen on the surface and higher inflammatory activation, whereas coating in HEPES buffer led to larger aggregates of collagen and reduced inflammation in softer environments. The data also points toward differences in local mechanosignaling.

Mechanotransduction studies commonly use collagen-coated PA gels to study changes to cellular adhesion, morphology, and phenotype due to the simplicity of technique and cost-effectiveness of the reagents. However, studies have noted differences in structure of surface-conjugated collagen, with some studies reporting thin fibrils of collagen,^36^ and others noting thick bundles.^37^ Such coating heterogeneity can cause discrepancies between different studies and can serve as a confounding factor. In addition, collagen self-assembly in vitro is a highly pH-sensitive process, and solvent pH determines the rate of fibrillogenesis and thickness of fibers formed.^38^ Earlier studies have shown that macrophages have a higher inflammatory response when cultured on purely elastic synthetic biomaterials, compared to viscoelastic ECM-based materials, demonstrating the complexity of considerations in the use of natural ECM biomaterials as cell culture substrates.^39^

The two examined conditions (at pH 3.4 and pH 8.5) result in distinct collagen microstructures that are known to exist—soluble, triple-helical at acidic conditions and fibrillar macromolecules at neutral and high pH.^20,23,38^ The different pH conditions are also typically used to deposit collagen on surfaces for in vitro cellular studies. In vivo, collagen conformation is important for normal tissue development,^40^ and differences in collagen integrity and ultrastructure can lead to pathological and inflammatory conditions.^41,42^ Changes in the fibrillar collagen microarchitecture (fiber thickness and pore size) have been shown to regulate myofibroblast differentiation and fibrosis through modulating local cellular mechanosignaling, independent of collagen concentration and bulk stiffness.^43^ Furthermore, low pH is observed in interstitial acidosis, which is associated with cancer and other conditions such as inflammation, ischemia, and metabolic disruptions and is likely altering the collagen architecture in these contexts. In the tumor microenvironment, the acidity of interstitial and intracellular pH is often reduced (as low as pH 5.6 in tumors)^44^ due metabolic activity. Collagen is a major component of the tumor microenvironment and can influence tumor and immune cell behaviors through various collagen receptors.^12^ In human breast cancer, infiltration of (tumor associated) macrophages is positively correlated with stiffness and TGF beta secretion and signaling,^45^ and increased collagen linearization and deposition leading to tissue inflammation.^45^ Fibrillar collagen levels and proteolysis are enhanced in tumor microenvironment, and denatured collagen acts as a strong chemoattractant for macrophages and mediates tumor progression.^46^ Given the macrophages’ roles in the diseases of aberrant ECM, such as fibroinflammation and tumorigenesis, understanding the roles of collagen conformation and distribution on stiffness-mediated inflammatory activation of macrophages is of paramount importance. Collagen conjugation performed at different pHs, and organization, could be crucial toward understanding these substrate–cell relationships, with potential to modulate and affect clinical outcome.^12,47^

Several cell receptors are known to bind collagen.^14,48^ Here, we found that the *Lair-1* (CD305) gene was the most highly expressed collagen receptor on cells when grown on both Col-HEP- and Col-AA-functionalized hydrogel surfaces compared to other known receptors such as integrins and DDRs. The LAIR-1 protein was expressed explicitly in cells cultured on softer Col-HEP-conjugated hydrogels (Figure 3B). One possible reason for Col-HEP surfaces suppressing inflammatory activation of macrophages (by LPS and IFNγ) is inhibition of inflammatory signaling mediated upon LAIR-1 ligation to its receptor,^49^ which may be facilitated by the pH-dependent conformation of collagen,^20^ in addition to substrate stiffness. On the other hand, uniform distribution and conformation of collagen on Col-AA surfaces might hinder cells to sense the substrate stiffness. Knocking down the *Lair-1* gene using siRNAs enhanced the secretion of inflammatory cytokines from cells, confirming its inhibitory function. However, the effect of knockdown was more pronounced in softer PA hydrogels. These results suggest that LAIR-1 receptor engagement is important for stiffness sensing and inflammatory activation in macrophages. However, the involvement of other receptors (e.g., integrins) in stiffness-mediated immunosuppression cannot be excluded.

We found that the solvent/solution in which collagen is solubilized and conjugated to a substrate can modulate the inflammatory response of macrophages and mask the effects of substrate stiffness. The BMDMs cultured on 1 kPa PA hydrogels attached with Col-AA expressed higher inflammatory genes and secreted more inflammatory cytokines than those in cells on Col-HEP. Even when the amount of conjugated collagen on the hydrogels was similar (Figure S3C), its distribution on the surface differed in Col-HEP and Col-AA conditions (Figures 4A,B and S3A). Earlier studies demonstrated that collagen I conformation was pH-dependent, which can thus impact the conformation of collagen conjugated on the surface.^20^ Collagen is more soluble at lower pH, yielding self-assembled trimers at low pH and increasingly larger fibrillar structures with increasing pH.^22,23,38,50^ Similarly in our study, collagen formed larger aggregates on the hydrogel surface when HEPES (pH 8.5) buffer was used to conjugate, and more homogeneous distribution in acetic acid solution, presumably of soluble collagen trimers (Figures 4A and S3A). A recent study also demonstrated that chronic inflammation can influence collagen ultrastructure and nanomechanical properties.^51^ However, how the change in collagen conformation and structure feeds back into inflammation is still unclear.

This homogeneous distribution of collagen on Col-AA surfaces may lead to greater adhesive interactions with macrophages, as visualized by increased vinculin staining, when compared to that in cells cultured on Col-HEP-conjugated surfaces (Figure 5A). It is possible that the homogeneous distribution leads to the exposure of epitopes or binding sites that directly interact with adhesive receptors such as integrins. As such, macrophage integrin expression has been linked to inflammatory activation in many contexts including cancer and inflammatory disease.^1^ We observed increased cell spreading on Col-AA compared to that in Col-HEP-conjugated surfaces, likely due to enhanced integrin-mediated focal adhesion (Figure 5C). These findings were in line with earlier observations wherein the focal adhesion protein vinculin was found to stabilize adhesion receptors, promote cell spreading, and transmit force at cell–cell and cell–matrix junctions.^52,53^ In addition, macrophage adhesion to its substrate is an important determinant of its inflammatory activation as our work has shown that short durations of adhesion or adhesion to ultralow binding surfaces significantly suppresses inflammatory activation.^17,54^ Increased cell spreading in Col-AA may have caused enhanced adhesion and translocation of YAP/TAZ into the nucleus, resulting in higher secretion of inflammatory cytokines. In addition, the fibrillar structure of Col-HEP-conjugated surfaces may discourage optimal cell–matrix adhesion and cell spreading, likely due to limited interactions with focal adhesions while promoting engagement of inhibitory receptor LAIR-1 with its ligands in the collagen; together, these effects could lead to suppressed inflammatory activation. We believe that understanding these interactions will help design biomaterials for improved wound healing and tissue repair and better understand the immunosuppressive environment of the tumor microenvironment.

## 5 Conclusions

The recognition of the ECM protein collagen to influence immune cell function has gained increased importance due to its immunomodulatory and therapeutic potential. Here, we analyzed the combined effects of substrate stiffness and solvent present to conjugate collagen I on the surface. Collagen distribution and conformation depended on the solvent used and ultimately determined the inflammatory activity of interacting macrophages. Further detailed molecular studies will be needed to determine how the changes in the supramolecular structure of collagen due to solvent pH affect the inflammatory activation of macrophages. Our studies are important because they show that seemingly minor variations in collagen substrate preparations for immunobiology studies can significantly alter critical innate cellular activation. Therefore, a better understanding of substrate surfaces, such as the effects of adsorbed proteins from sera, their surface distributions, and the resulting local surface topologies and molecular conformations, on immune cell responses could inform the design of materials used in medicine.

## Supporting Information Available

Primer sequences for qPCR, inflammatory activation of macrophages at alternative conditions, collagen distribution on substrates, SEM images, and raw data for Western blots (PDF)
