# Supplementary material for: Modulation of Stiffness-Dependent Macrophage Inflammatory Responses by Collagen Deposition
Source: ACS Biomater Sci Eng. 2024 Mar 11;10(4):2212–23. doi: 10.1021/acsbiomaterials.3c01892 (PMC11005009; doi:10.1021/acsbiomaterials.3c01892)

Supplementary Information

Modulation of Stiffness-Dependent Macrophage Inflammatory Responses by Collagen Deposition

Vijaykumar S. Meli[1,2,3], Andrew T. Rowley[3], Praveen K. Veerasubramanian[1,2], Sara E. Heedy[3], Wendy F. Liu *[1,2,3,4,5] and Szu-Wen Wang* [1,3,5,6]

[1] Department of Biomedical Engineering
[2] UCI Edwards Lifesciences Foundation Cardiovascular Innovation and Research Center
[3] Department of Chemical and Biomolecular Engineering
[4] Department of Molecular Biology and Biochemistry
[5] Institute for Immunology
[6] Chao Family Comprehensive Cancer Center

University of California Irvine
Irvine, CA 92697

* To whom correspondence may be addressed:
WFL: wendy.liu@uci.edu
SWW: wangsw@uci.edu

Gene	Primer sequence (5'-3')
**LAIR1-1F**	TCCTCCTTTGTCTTTCCGCC
**LAIR1-1R**	CAGGAAGCCTGTCATCTGCA
**TnfF**	CCCACGTCGTAGCAAACCACC
**TnfR**	TCGGGGCAGCCTTGTCCCTT
**IL6F**	CTGCAAGAGACTTCCATCCAGTT
**IL6R**	GAAGTAGGGAAGGCCGTGG
**Nos2F**	GAATCTTGGAGCGAGTTGTGG
**Nos2R**	TTGTACTCTGAGGGCTGACAC
**Ddr1F**	ACAACAGCCAGTGACGTTTG
**Ddr1R**	ATCAGCTCATACAGGGTCTGTG
**GPVIF**	TTGTGGTTACTGGACTCTCTGC
**GPVIR**	TTGGCATAGTGCTGATGAGC
**Itgb1F**	TGGACAATGTCACCTGGAAA
**Itgb1R**	GTGTGCCCACTGCTGACTTA
**GapdhF**	GTCAAG CTCATTTCCTGGTAT
**GapdhR**	TCTCTTGCTCAGTGTCCTTGC

Table S1: Primer sequences used for qPCR in this study.

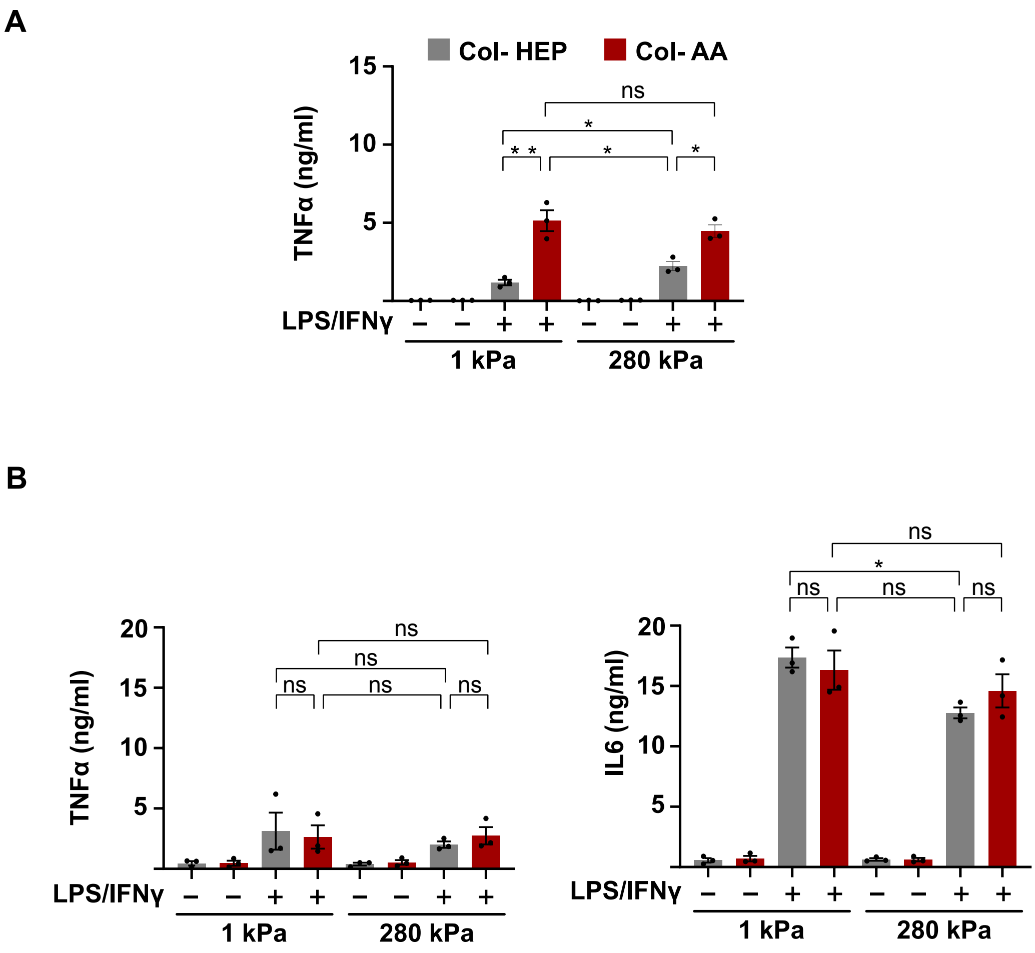

Figure S1: Solvent system and collagen conjugation determine the inflammatory activation of macrophages. (A) Secretion of TNF-α by THP-1 monocytes differentiated to macrophages using PMA for 42 h on PA hydrogels of 1 and 280 kPa coated with collagen-HEPES (Col-HEP) and collagen –acetic acid (Col-AA) and stimulated with M1 cytokines for 24 h. (B) Secretion of TNFα (left) and IL6 (right) by mouse BMDMs after 24 h of adhesion onto uncoated PA hydrogels of 1 and 280 kPa and stimulated with M1 cytokines for 24 h. The values are the mean ± S.E.M. from at least three individual donors assessed by one-way ANOVA with Tukey's multiple comparisons. * *P<0.05, **P<0.005.*

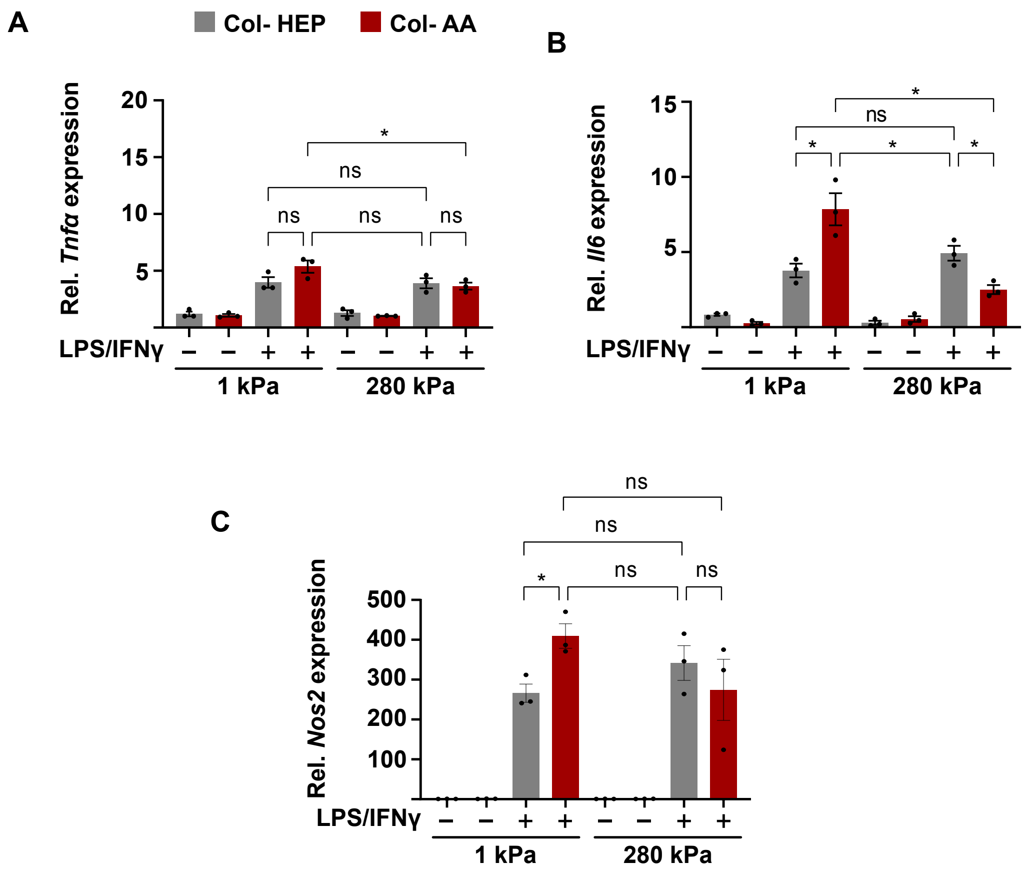

Figure S2: Solvent system used for collagen conjugation on Col-HEP and Col-AA PA hydrogels affects inflammatory activation of macrophages. Relative gene expression of *Tnf*α (A), *Il-6* (B)*,* and *Nos2* (C) assessed by quantitative PCR in BMDMs cultured on Col-HEP and Col-AA for 24 h and stimulated with M1 cytokines for 24 h. The values are the mean ± S.E.M. from at least individual donors assessed by one-way ANOVA with Tukey's multiple comparisons. * *P<0.05, **P<0.005.*

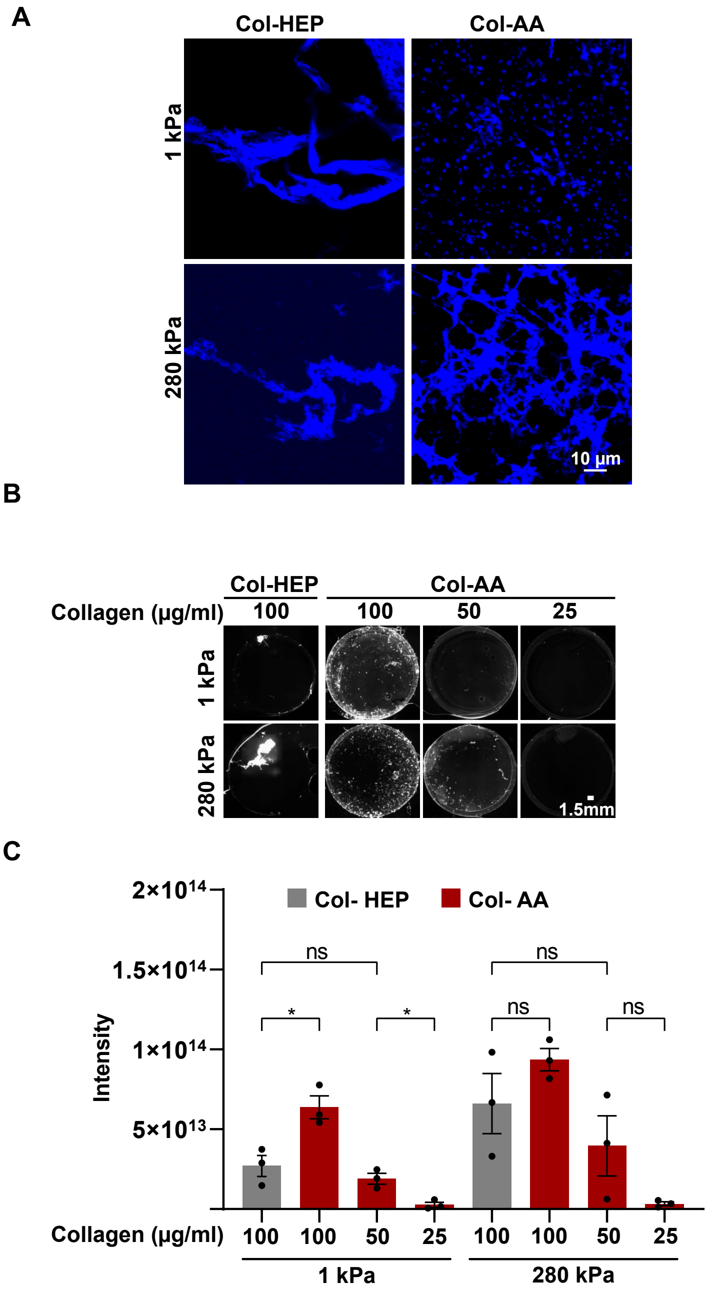

Figure S3: Collagen conjugating solvent determines the collagen distribution on hydrogel surfaces. (A) Fluorescence confocal images of hydrogel surfaces coated with Cy5 labeled collagen. (B) Fluorescence images of the entire PA hydrogels of 1 and 280 kPa coated overnight at 4°C with different amounts of Cy5 labeled collagen on Col-HEP and Col-AA surfaces. (C) Quantification of intensity of Cy5-collagen described in panel (B), at deposition concentrations of 100, 50, and 25 µg/ml. The values are the mean ± S.E.M. from at least three individual donors assessed by pair-wise comparisons using two-tailed Student's *t* test for collagen intensities. * *P<0.05.*

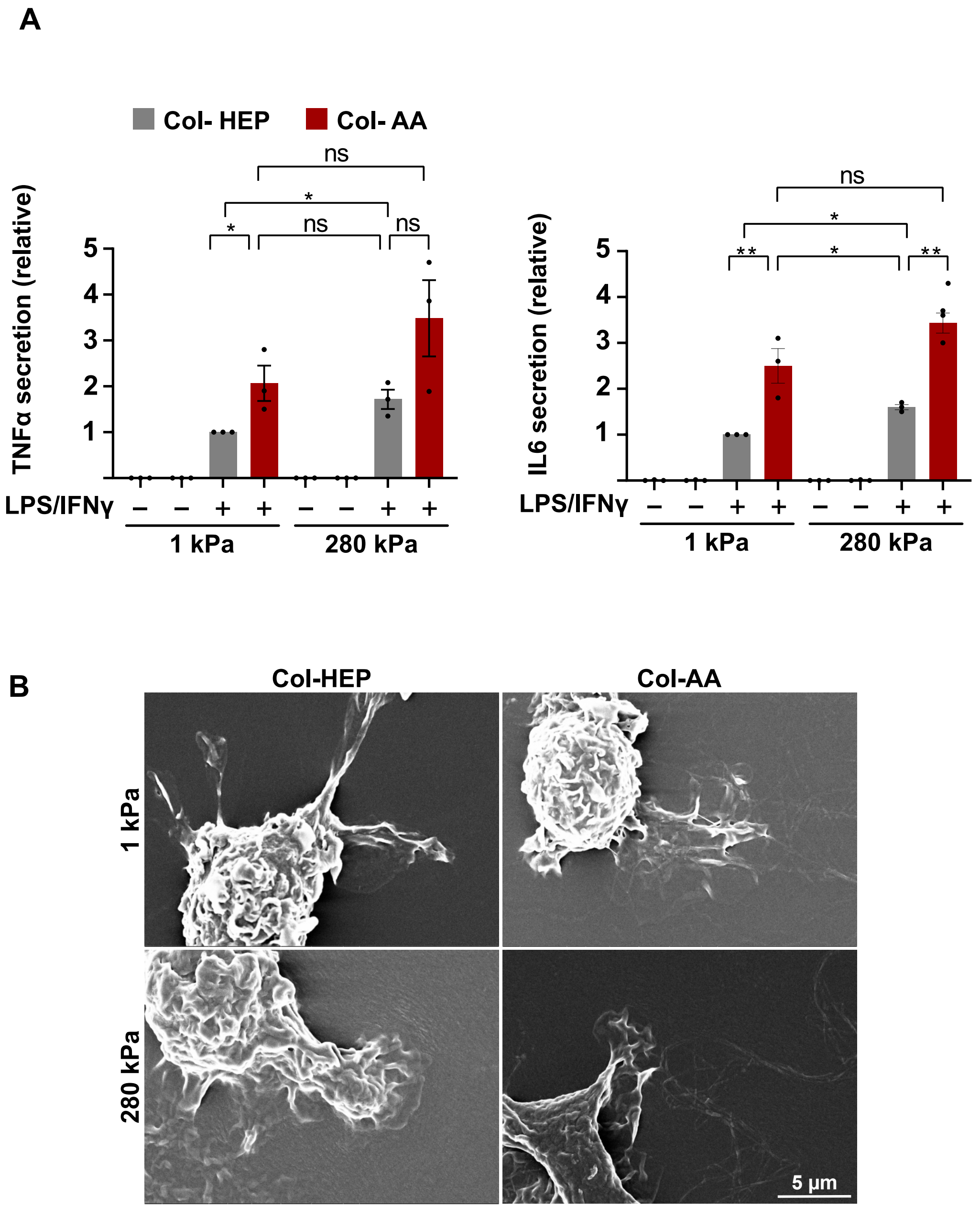

Figure S4: Solvent system and collagen conjugation determine the inflammatory activation of macrophages. (A) Secretion of TNF-α and IL-6 by mouse BMDMs after 24 h of adhesion onto PA hydrogels of 1 and 280 kPa conjugated with 100 µg /ml of collagen on Col-HEP and 50 µg /ml of collagen on Col-AA surfaces and stimulated with M1 cytokines for 24 h. The values are the mean ± S.E.M. from at least three individuals assessed by one-way ANOVA with Tukey's multiple comparisons. *$P<0.05$, **$P<0.005$. (B) Scanning electron microscopy images of PA hydrogel surfaces conjugated with collagen and cultured with BMDMs for 24 h.

Figure S5. Supporting raw data of immunoblots. Uncropped western immunoblot images for Fig. 2B data for iNOS and GAPDH control, on soft and stiff substrates (n=3 replicates). For each gel, the molecular weight standards shown (left) are from white light images of the corresponding or representative nitrocellulose membranes after blotting.

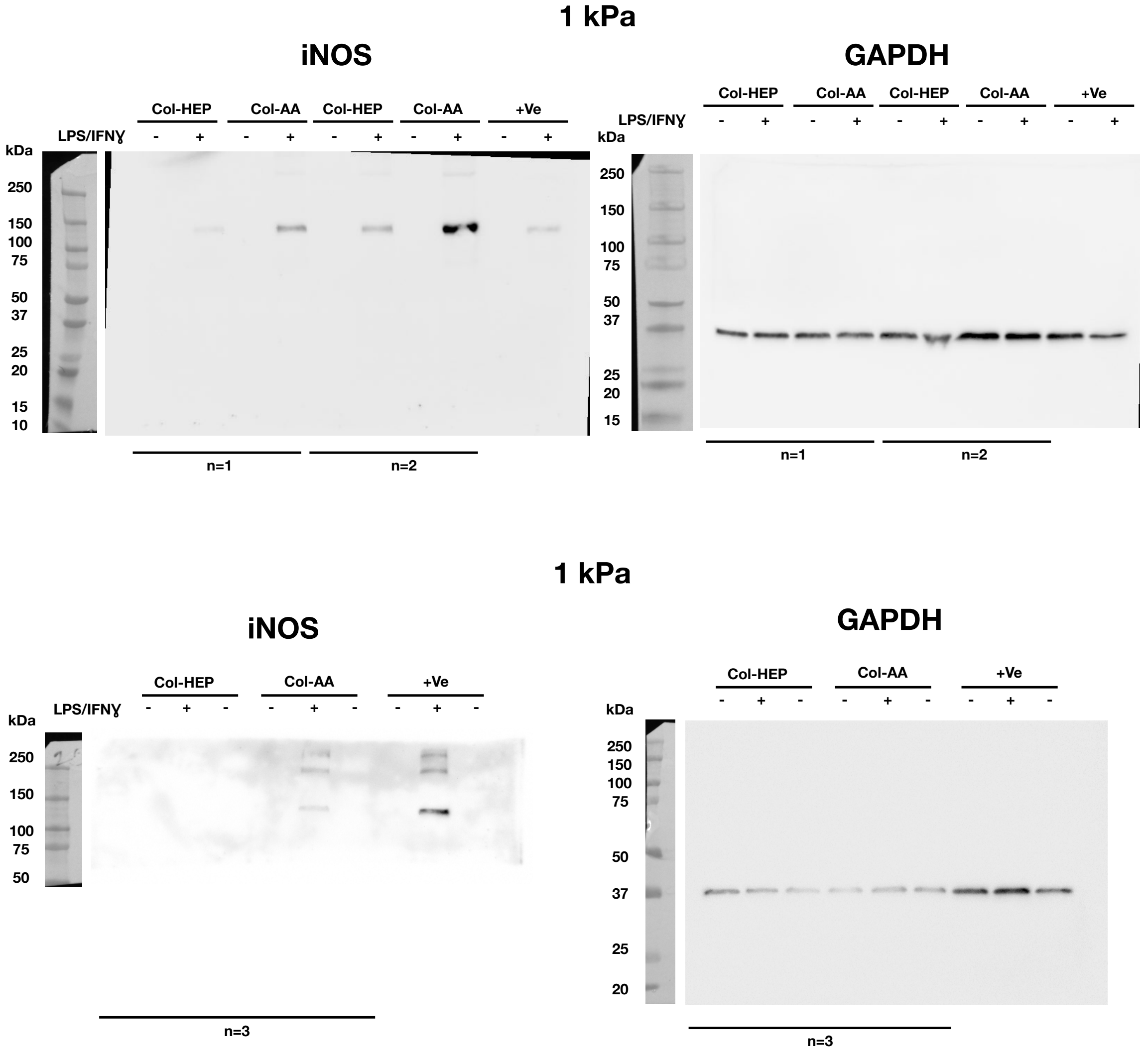

280 kPa

iNOS

GAPDH

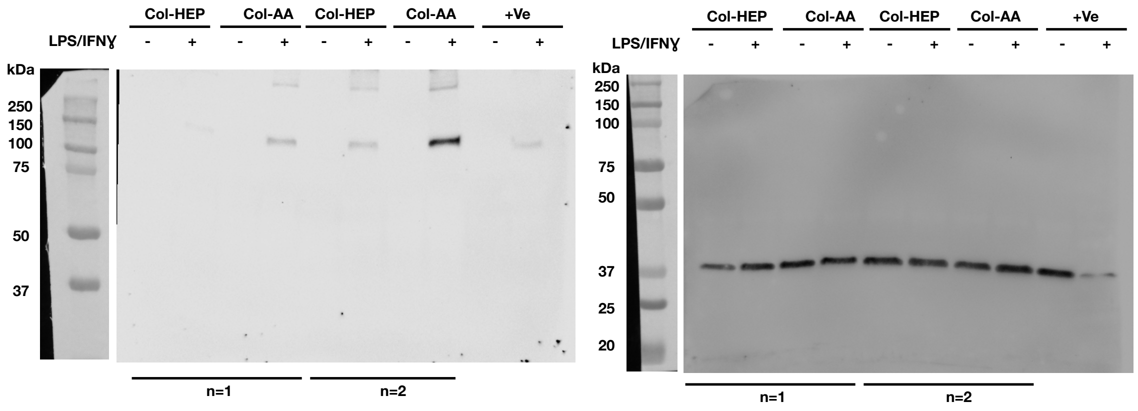

280 kPa

iNOS

GAPDH

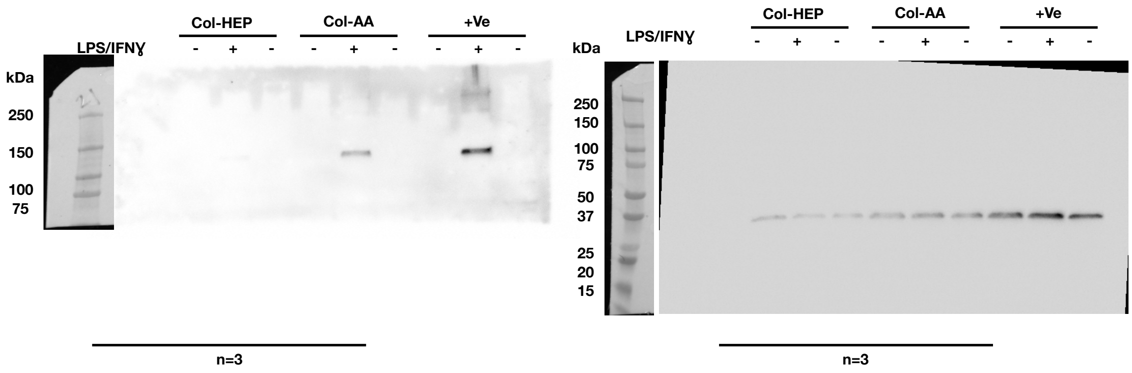

Supplement: Supplementary file 1 — ab3c01892_si_001.pdf [file ab3c01892_si_001.pdf]
